# Interpretable machine learning for predicting major amputation risk in hospitalized diabetic foot ulcer patients: a single-center study with temporal external validation

**DOI:** 10.3389/fendo.2026.1821550

**Published:** 2026-05-22

**Authors:** Meiling Zou, Shang Ju

**Affiliations:** Department of Peripheral Vascular Surgery, Dongzhimen Hospital, Beijing, China

**Keywords:** diabetic foot ulcer, explainable model, hospitalization, major amputation, random forest, risk prediction, Shapley additive explanations, temporal validation

## Abstract

**Background:**

Diabetic foot ulcers are a leading cause of non-traumatic lower-limb amputation, but early identification of inpatients at high risk of major amputation remains challenging.

**Methods:**

We retrospectively reviewed consecutive admissions for diabetic foot ulcers at a single center, developing models in a 2019–2020 cohort and temporally validating them in a later 2024 cohort. The outcome was in-hospital major lower-extremity amputation above the ankle. Candidate predictors were routinely available admission variables within 24 hours, including comorbidities, bedside limb/ulcer assessment, and standard laboratory tests. We compared logistic regression, elastic net, random forest, and extreme gradient boosting models and used Shapley additive explanations to provide patient-level interpretability.

**Results:**

The random forest model showed the best overall discrimination, with an area under the receiver operating characteristic curve of 0.977 in internal testing and 0.984 in temporal validation, and acceptable calibration. The most influential predictors reflected limb perfusion and infection severity and included perfusion grade, ankle–brachial index, maintenance dialysis, white blood cell count, C-reactive protein, and prior minor amputation.

**Conclusions:**

An explainable admission-data model can support early inpatient risk stratification for major amputation in diabetic foot ulcer patients and may help prioritize timely multidisciplinary care.

## 1 Introduction

Diabetic foot ulcer (DFU) represents a severe manifestation of diabetes and is frequently associated with adverse limb outcomes. The lifetime risk of developing DFU among individuals with diabetes has been reported to be approximately 34% (1). DFU is a major driver of lower-extremity amputation (LEA) and remains a leading contributor to non-traumatic LEA globally (2). Epidemiological studies suggest that roughly one in five patients with DFU eventually undergoes amputation, underscoring the substantial clinical and health-system burden of this condition (2–5).

In routine DFU care, clinicians commonly use structured grading systems—such as the diabetic ulcer severity score, the Meggitt–Wagner classification, and the University of Texas wound classification—to describe wound severity and support treatment planning (6–8). Although these schemes can partially predict major amputation risk, none is universally accepted as a gold standard (9). A key limitation is that they focus mainly on wound-related features and do not integrate multi-domain prognostic information (for example, demographics, comorbidities, laboratory indices, prior medical history, and limb/foot characteristics) into a single framework (10, 11). As a result, risk discrimination for major amputation may remain suboptimal in real-world hospitalized DFU populations.

Diabetic foot ulcers are clinically heterogeneous, and their management generates multimodal information across disciplines, including endocrinology, imaging, and surgery. Machine learning (ML) and other artificial intelligence (AI) approaches can learn patterns from such data to anticipate healing trajectories, estimate amputation risk, and support individualized treatment planning using routinely collected clinical variables (12, 13). Accordingly, several studies have explored ML-based prediction of major amputation in DFU populations (14). However, model complexity can hinder bedside adoption when clinicians require transparent reasoning to trust and act on risk estimates (15, 16). The limited explainability of many ‘black-box’ models also restricts clinical translation (17). To address these barriers, we paired ML modeling with Shapley additive explanations (SHAP), which quantify how each feature contributes to an individual patient’s predicted risk (18).

Compared with minor amputation, major amputation is typically associated with greater loss of mobility and a larger decrement in health-related quality of life (19). Nevertheless, limited interpretability remains a practical barrier to incorporating many ML models into clinical decision-support workflows (20). Therefore, using inpatient data from our medical center, we developed and compared multiple ML models to predict in-hospital major amputation risk and selected an optimal model for explainability analyses. We used SHAP to visualize and interpret the model and to identify key prognostic factors influencing outcomes (18, 21). Our aim was to develop and temporally validate an interpretable model that estimates the risk of in-hospital major amputation among adults admitted with DFU, and to provide transparent explanations to support early risk stratification and targeted inpatient management (22–24).

## 2 Materials and methods

**Figure 1:**
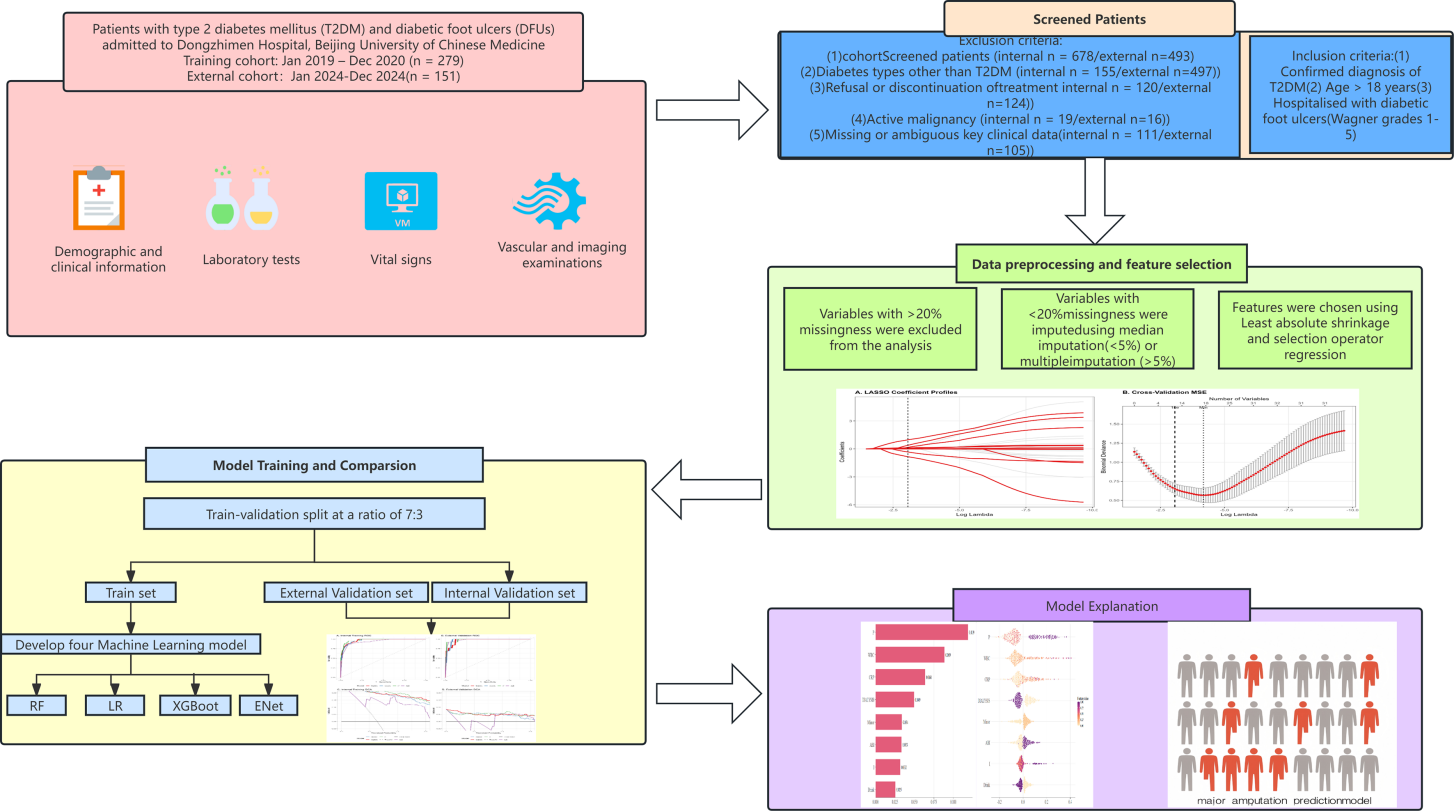
Study workflow for cohort screening, model development, validation, and interpretation. Flow diagram showing patient screening and eligibility assessment for the derivation and temporal validation cohorts, followed by data preprocessing, feature selection, model development, performance evaluation, and model interpretation. DFU, diabetic foot ulcer; ENet, elastic net; LASSO, least absolute shrinkage and selection operator; LR, logistic regression; RF, random forest; T2DM, type 2 diabetes mellitus; XGBoost, extreme gradient boosting.

This retrospective cohort study developed, compared, and interpreted prediction models for in-hospital major amputation among adults hospitalized with diabetic foot ulcers. After data extraction and preprocessing, we selected candidate predictors available within 24 hours of admission, performed feature selection, trained multiple algorithms, and evaluated performance in an internal test set and an independent temporal validation cohort. Model interpretability was assessed using Shapley additive explanations SHAP to quantify feature contributions at both the cohort global and patient local levels. The overall workflow is summarized in **Figure 1**.

### 2.1 Study population and data acquisition

Consecutive adult patients age ≥18 years admitted to our hospital between January 2019 and December 2020 with diabetic foot ulcer and Wagner grade 1–5 were screened for inclusion (10). Eligibility criteria were confirmed type 2 diabetes mellitus and a diagnosis of diabetic foot ulcer on admission. Exclusion criteria were diabetes types other than type 2, refusal or discontinuation of inpatient treatment, active malignancy, and substantial missing clinical data or unclear outcome documentation. After applying these criteria, 279 patients were included in the derivation cohort.

A temporal external validation cohort was assembled from the same center using admissions between January 2024 and December 2024 and applying identical inclusion and exclusion criteria. The validation cohort comprised 151 patients. The study was approved by the institutional ethics committee, and informed consent was waived because the analysis used de-identified retrospective data and did not affect clinical care.

### 2.2 Outcome definition

The primary outcome was in-hospital major lower-extremity amputation (25). Major amputation was defined as any amputation performed above the ankle joint, including below-knee and above-knee amputations. Procedures below the ankle (for example, toe disarticulation, ray amputation, or forefoot amputation) were classified as minor amputation and were not counted as primary outcome events; however, prior minor amputation history was considered as a candidate predictor.

Amputation decisions were made by vascular surgeons or orthopedic foot-and-ankle surgeons based on a comprehensive assessment of limb ischemia, extent of infection, tissue loss, functional prognosis, and overall clinical status (23, 24). Outcome events were verified using operative reports, anesthetic records, and discharge summaries, supplemented by preoperative imaging when needed to confirm the level of amputation (26, 27). Two investigators independently adjudicated outcome status using predefined criteria, with discrepancies resolved by consensus or consultation with a third investigator. However, we acknowledge that these procedures cannot fully eliminate the influence of clinical judgment on whether limb salvage is pursued and at what level major amputation is performed. Therefore, the study endpoint was defined pragmatically as in-hospital major amputation during the index hospitalization, including both patients undergoing early major amputation after admission assessment and those requiring major amputation later during hospitalization after further evaluation and/or attempted limb-salvage management. In DFU, limb outcomes are shaped not only by local wound severity, ischemia, and infection, but also by broader systemic vulnerability, including chronic tissue injury and nutritional and inflammatory status (28).

### 2.3 Candidate predictors

Candidate predictors were derived from routine clinical information and laboratory tests obtained within the first 24 hours after admission, ensuring availability for early inpatient risk stratification (27, 29). No variables derived from the subsequent inpatient course, operative planning, discharge documentation, or outcome adjudication were entered into model development.Variables included demographic characteristics (age, sex, duration of diabetes), comorbidities (for example, hypertension, hyperlipidemia, cerebrovascular disease, coronary heart disease, peripheral neuropathy, peripheral arterial disease, renal insufficiency, and maintenance dialysis), infection-related factors (osteomyelitis and multidrug-resistant infection), and lifestyle and disease-course factors (smoking, alcohol use, recurrent ulceration, and prior minor amputation) (30). Foot- and limb-specific variables included ankle–brachial index ABI, rest pain, and ulcer severity assessed using the PEDIS system (perfusion, extent/depth, infection, and sensation), which was treated as an ordinal categorical measure (31). The PEDIS classification was handled by treating its individual components as separate candidate predictors rather than as a single composite score. Only the components retained through the selection procedure were included in the final model. ABI was dichotomized using the conventional threshold of <0.9 to reflect clinically relevant peripheral arterial disease and to improve interpretability in bedside risk stratification. We acknowledge, however, that this approach may reduce granularity and obscure risk differences across more severe ABI ranges. Laboratory parameters reflected systemic inflammation, nutritional status, glycemic control, and coagulation–fibrinolysis and included white blood cell count, neutrophil percentage, hemoglobin, platelet count, C-reactive protein, fibrinogen, D-dimer, fasting plasma glucose, glycated hemoglobin, serum creatinine, and serum albumin (23). All candidate predictors were initially considered, and least absolute shrinkage and selection operator LASSO regression was used to identify the most informative variables for model development (30, 32).

### 2.4 Data preprocessing and feature selection

Variables with more than 20% missing data were excluded before model development to reduce instability in feature selection and minimize reliance on extensive imputation in this retrospective dataset. This threshold was selected as a pragmatic balance between retaining potentially relevant predictors and avoiding unreliable estimation for variables with limited observed data. However, we acknowledge that missingness in clinical datasets may itself be informative, as the absence of specific laboratory or vascular assessments may reflect clinician judgment, illness severity, or local resource a vailability. For variables with less than 5% missing values, missing entries were imputed using the median. When missingness exceeded 5%, we used random-forest-based multiple imputation (33). To reduce model dimensionality and mitigate overfitting, we performed least absolute shrinkage and selection operator LASSO regression on the training partition of the derivation cohort. LASSO shrinks coefficients toward zero under an L1 penalty, and the degree of shrinkage is governed by the tuning parameter lambda. We selected lambda using 10-fold cross-validation; lambda_min minimized the cross-validation error, and lambda_1se was the largest value within one standard error of the minimum (34). To favor parsimony and generalizability, we selected lambda_1se and retained eight predictors for subsequent model development.

### 2.5 Model development

We developed four models representing distinct modeling paradigms: logistic regression LR, elastic net regression ENet, random forest RF, and extreme gradient boosting XGBoost. Including both penalized linear models and nonlinear ensemble learners allowed us to compare performance across approaches with different inductive biases.

The patients were randomly split into a training dataset and a testing dataset in a 7:3 ratio. Predictor selection was performed using the training set only prior to model fitting. Subsequently, we developed a comprehensive suite of four machine learning algorithms to build robust predictive models. These algorithms were carefully selected to encompass a broad range of approaches, from traditional statistical methods to advanced ensemble techniques and neural networks. The implemented models included: Elastic Net ENet, Logistic Regression, Random Forest RF, and eXtreme Gradient Boosting XGBoost.Model training employed ten-fold cross-validation on the training set and Hyperparameters, the predictive performance of each model was evaluated using ROC curves in both the training and testing datasets. The prediction model with the best performance on the testing set was ultimately selected. The interpretation of the predictive model was performed using the SHAP (Shapley Additive exPlanations) method, which accurately computes the contribution and impact of each feature on the final prediction. We calculated SHAP values using the fastshap package in R.

For global interpretability, we quantified overall feature importance using the mean absolute SHAP value (mean|SHAP|) and visualized the ranking with horizontal bar charts. To summarize effect patterns, we generated SHAP summary figures: for continuous predictors, beeswarm-type plots were used to depict the distribution of SHAP values for each variable; for categorical predictors, SHAP values were displayed by category level using box-and-whisker plots with overlaid points. These SHAP outputs offer non-trivial insight into how individual predictors influence the model’s predictions.

Within the derivation cohort, patients were randomly split into a training set and a held-out internal test set in a 7:3 ratio. Feature selection LASSO was performed using the training set only, and final models were fitted using the selected predictors. Hyperparameters were tuned by 10-fold cross-validation on the training set. Discrimination was assessed using the receiver operating characteristic (ROC) curve and its area under the curve, and classification metrics were computed at an optimized decision threshold. For model explainability, SHAP values were computed to quantify the contribution of each predictor to the predicted risk; SHAP calculations were implemented in R using the fastshap package.

### 2.6 Performance evaluation and interpretability

Model performance was evaluated using a held-out internal test set and an independent temporal external validation cohort. Discrimination was quantified via the area under the ROC curve AUC, sensitivity, specificity, F1-score, accuracy, and balanced accuracy. The 95% confidence intervals CIs for the AUCs were calculated using the bootstrap method with 2,000 resamples with replacement to provide robust interval estimates. To assess calibration and overall probabilistic accuracy, we utilized the Brier score, log loss, calibration intercept, calibration slope, and the Hosmer-Lemeshow goodness-of-fit test, supplemented by visual inspection of calibration curves. To derive a clinically usable operating point, we selected the decision threshold via tuned-threshold cross-validation 10-fold cross-validation with 100 repeats), optimizing the F1-score to balance sensitivity and specificity. For explainability, SHAP values were computed to estimate each predictor’s contribution to the predicted risk at both the patient level and the cohort level. As a complementary local approach, we also applied local interpretable model-agnostic explanations (LIME) (35).

### 2.7 Statistical analysis

Descriptive statistics were performed in R (version 4.2.2). Categorical variables are presented as counts (percentages) and compared using the chi-square test. Continuous variables are summarized as mean (standard deviation) when approximately normally distributed or as median (interquartile range) otherwise, with group comparisons using the independent-samples t-test or the Mann–Whitney U test as appropriate. In addition to statistical significance testing, we calculated the standardized mean difference (SMD) to assess the magnitude of differences in baseline characteristics between the amputation and non-amputation groups. An SMD > 0.10 was considered indicative of a potentially meaningful difference, independent of sample size.All tests were two-sided, and statistical significance was defined as p < 0.05.

## 3 Results

### 3.1 Demographic and baseline clinical characteristics

Baseline characteristics of the derivation cohort are summarized in **Table 1**. The derivation cohort included 279 hospitalized patients with diabetic foot ulcers; 71 25.4%) underwent major amputation during hospitalization. Baseline age, duration of diabetes, and sex distribution did not differ significantly between patients with and without major amputation (all p > 0.05, SMD < 0.10). Peripheral arterial disease PAD and maintenance dialysis were more frequent in the major amputation group, showing meaningful effect magnitudes (PAD: 83.1% vs 68.3%, p = 0.024, SMD = 0.35; dialysis: 74.6% vs 36.1%, p < 0.001, SMD = 0.83), and alcohol use was also more common (73.2% vs 45.7%, p < 0.001, SMD = 0.57). In contrast, hypertension, cerebrovascular disease, and peripheral neuropathy were more frequent in the non-amputation group (all p < 0.01, SMD > 0.40). According to the PEDIS classification (30), patients in the major amputation group had more severe ischemia, deeper tissue involvement, and more advanced infection, with higher proportions of P3 (severe ischemia), D3–4 (deep tissue, bone, or joint involvement), and I3–4 (severe or life-threatening infection) (all p < 0.001). Laboratory findings suggested greater systemic inflammation and a prothrombotic profile in the major amputation group. Notably, several inflammatory markers demonstrated large effect sizes between the groups, including higher white blood cell count (SMD = 1.18), neutrophil percentage (SMD = 1.10), and C-reactive protein (SMD = 1.12) (all p < 0.001). Other elevated markers included platelet count (SMD = 0.71), fibrinogen (SMD = 0.43), and D-dimer (SMD = 0.49) (all p < 0.01), together with lower hemoglobin (SMD = 0.80) and albumin (SMD = 0.57) (both p < 0.001). Fasting plasma glucose, glycated hemoglobin, and serum creatinine did not differ significantly between groups.

**Table 1:** Characteristics of the derivation cohort patients at first admission.

Variables	Total (n = 279)	Non-amputation (n = 208)	Amputation (n = 71)	P value	SMD
Demographics					
Age (years)	65.76 ± 10.40	66.01 ± 10.47	65.03 ± 10.21	0.488	0.09
Duration of diabetes (years)	16.14 ± 8.63	16.12 ± 8.81	16.20 ± 8.14	0.944	0.01
Male, n (%)	180 (64.5%)	132 (63.5%)	48 (67.6%)	0.627	0.09
Medical history and clinical characteristics					
Hypertension, n (%)	150 (53.8%)	125 (60.1%)	25 (35.2%)	<0.001	0.52
Hyperlipidemia, n (%)	166 (59.5%)	125 (60.1%)	41 (57.7%)	0.835	0.05
Cerebrovascular disease, n (%)	124 (44.4%)	103 (49.5%)	21 (29.6%)	0.005	0.41
Coronary heart disease, n (%)	134 (48.0%)	102 (49.0%)	32 (45.1%)	0.660	0.08
Peripheral neuropathy, n (%)	142 (50.9%)	118 (56.7%)	24 (33.8%)	0.001	0.47
Ankle-brachial index <0.9, n (%)	151 (54.1%)	131 (63.0%)	20 (28.2%)	<0.001	0.73
History of minor amputation, n (%)	89 (31.9%)	82 (39.4%)	7 (9.9%)	<0.001	0.74
Smoking, n (%)	150 (53.8%)	104 (50.0%)	46 (64.8%)	0.088	0.30
Alcohol consumption, n (%)	147 (52.7%)	95 (45.7%)	52 (73.2%)	<0.001	0.57
Renal insufficiency, n (%)	114 (40.9%)	79 (38.0%)	35 (49.3%)	0.125	0.23
Dialysis, n (%)	128 (45.9%)	75 (36.1%)	53 (74.6%)	<0.001	0.83
Recurrent ulcer, n (%)	176 (63.1%)	143 (68.8%)	33 (46.5%)	0.001	0.46
Rest pain, n (%)	125 (44.8%)	104 (50.0%)	21 (29.6%)	0.004	0.42
Osteomyelitis, n (%)	155 (55.6%)	114 (54.8%)	41 (57.7%)	0.770	0.06
Peripheral artery disease, n (%)	201 (72.0%)	142 (68.3%)	59 (83.1%)	0.024	0.35
Multidrug-resistant infection, n (%)	141 (50.5%)	105 (50.5%)	36 (50.7%)	1.000	0.00
Laboratory data					
White blood cell count (×10^9^/L)	10.39 ± 5.77	8.62 ± 3.62	15.55 ± 7.57	<0.001	1.18
Hemoglobin (g/L)	110.93 ± 21.29	115.25 ± 18.60	98.28 ± 23.64	<0.001	0.80
Platelet count (×10^9^/L)	300.06 ± 117.23	277.84 ± 98.10	365.14 ± 142.69	<0.001	0.71
Neutrophil percentage (%)	72.09 ± 12.51	68.97 ± 11.63	81.23 ± 10.36	<0.001	1.10
C-reactive protein (mg/L)	57.90 ± 68.05	38.91 ± 52.85	113.56 ± 76.92	<0.001	1.12
Fasting plasma glucose (mmol/L)	8.58 ± 3.94	8.40 ± 3.60	9.11 ± 4.80	0.259	0.17
Creatinine (μmol/L)	96.95 ± 124.19	93.54 ± 114.66	106.94 ± 149.12	0.491	0.10
Albumin (g/L)	32.92 ± 5.59	33.70 ± 5.53	30.62 ± 5.13	<0.001	0.57
Hemoglobin A1c (%)	8.19 ± 1.92	8.24 ± 1.98	8.04 ± 1.74	0.439	0.11
Fibrinogen (g/L)	5.11 ± 2.90	4.72 ± 1.69	6.28 ± 4.81	0.009	0.43
D-dimer (μg/mL)	455.66 ± 646.59	377.25 ± 636.68	685.38 ± 624.38	0.001	0.49

Data are presented as mean ± standard deviation or n (%). Major amputation was defined as any amputation above the ankle. Grades P (perfusion), D (depth/tissue loss), I (infection), and S (sensation) follow the PEDIS classification system.

### 3.2 Variable selection using LASSO regression

**Figure 2:**
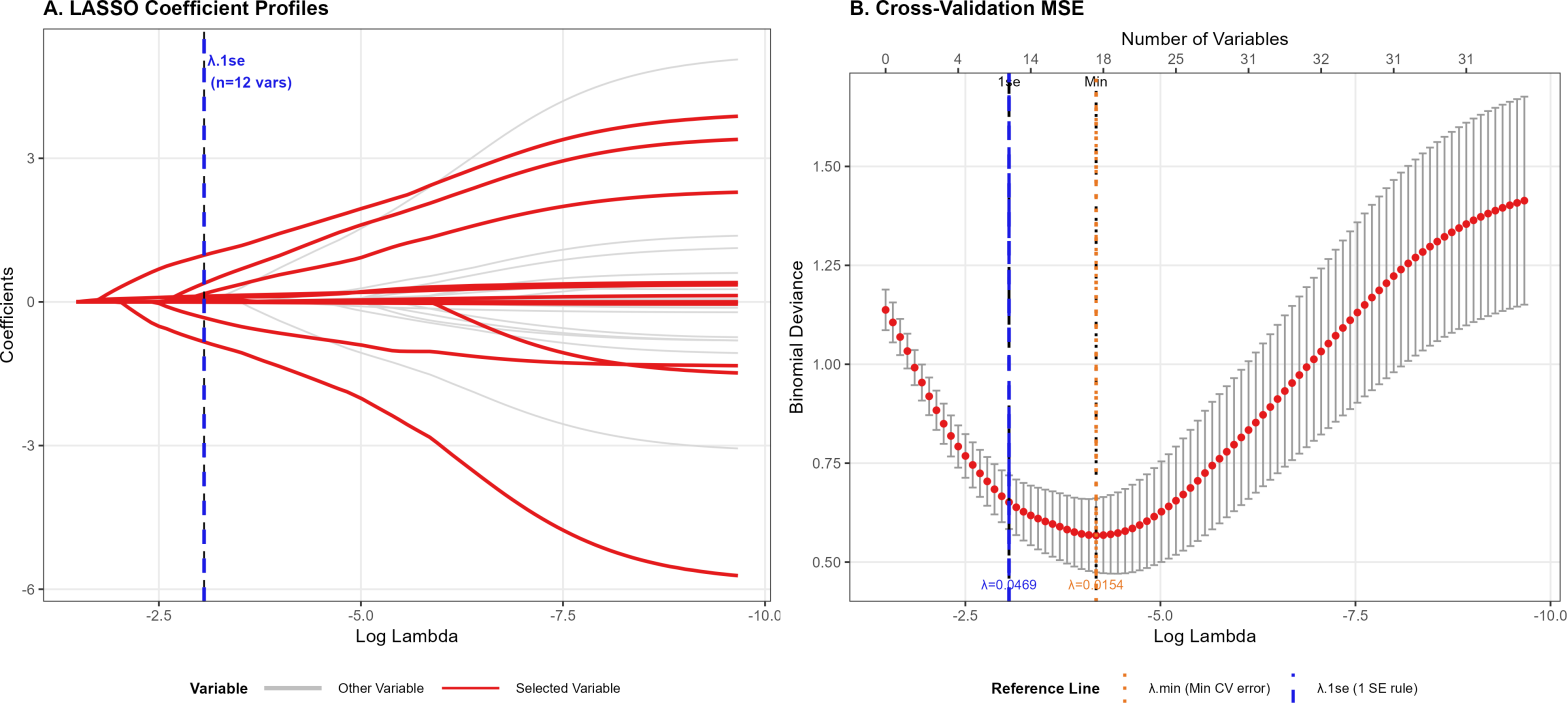
LASSO regression analysis was used to select potential variables. Two-panel figure showing **(A)** LASSO coefficient trajectories across log lambda values, with selected variables highlighted, and **(B)** cross-validation error across lambda values, with reference lines indicating λ_min and λ_1se. CV, cross-validation; LASSO, least absolute shrinkage and selection operator; MSE, mean squared error.

To reduce dimensionality and limit multicollinearity, we applied LASSO-penalized logistic regression to all candidate predictors (32). The penalty parameter lambda was selected using cross-validation across candidate values, with coefficient paths and cross-validated error summarized in **Figure 2**. We used the one-standard-error rule λ_1se to favor a parsimonious model with improved generalizability. At λ_1se, eight predictors retained non-zero coefficients and were carried forward for model development.

Two candidate penalty values were identified from cross-validation: λ_min (the λ yielding the minimum cross-validated error) and λ_1se (the largest λ within one standard error of the minimum error) (**Figure 2**). To preserve predictive performance while improving parsimony and generalizability, λ (1).se was selected as the final penalty. At this λ, eight key predictors retained non-zero coefficients (highlighted in red in **Figure 2**) 36. These predictors broadly reflect limb perfusion and tissue destruction, infection severity, and systemic inflammatory/nutritional status. The selected eight features were subsequently used as inputs for downstream machine-learning models and the conventional regression comparator to reduce overfitting and enhance robustness.

### 3.3 Model development and performance comparison

Using the eight predictors selected by LASSO, we trained four models—LR, ENet, RF, and XGBoost—and compared their performance in the internal test set and the temporal external validation cohort. Hyperparameters were tuned by cross-validation, and discrimination was summarized by the area under the ROC curve together with accuracy, sensitivity, specificity, and F1-score at the selected decision threshold.

**Figure 3:**
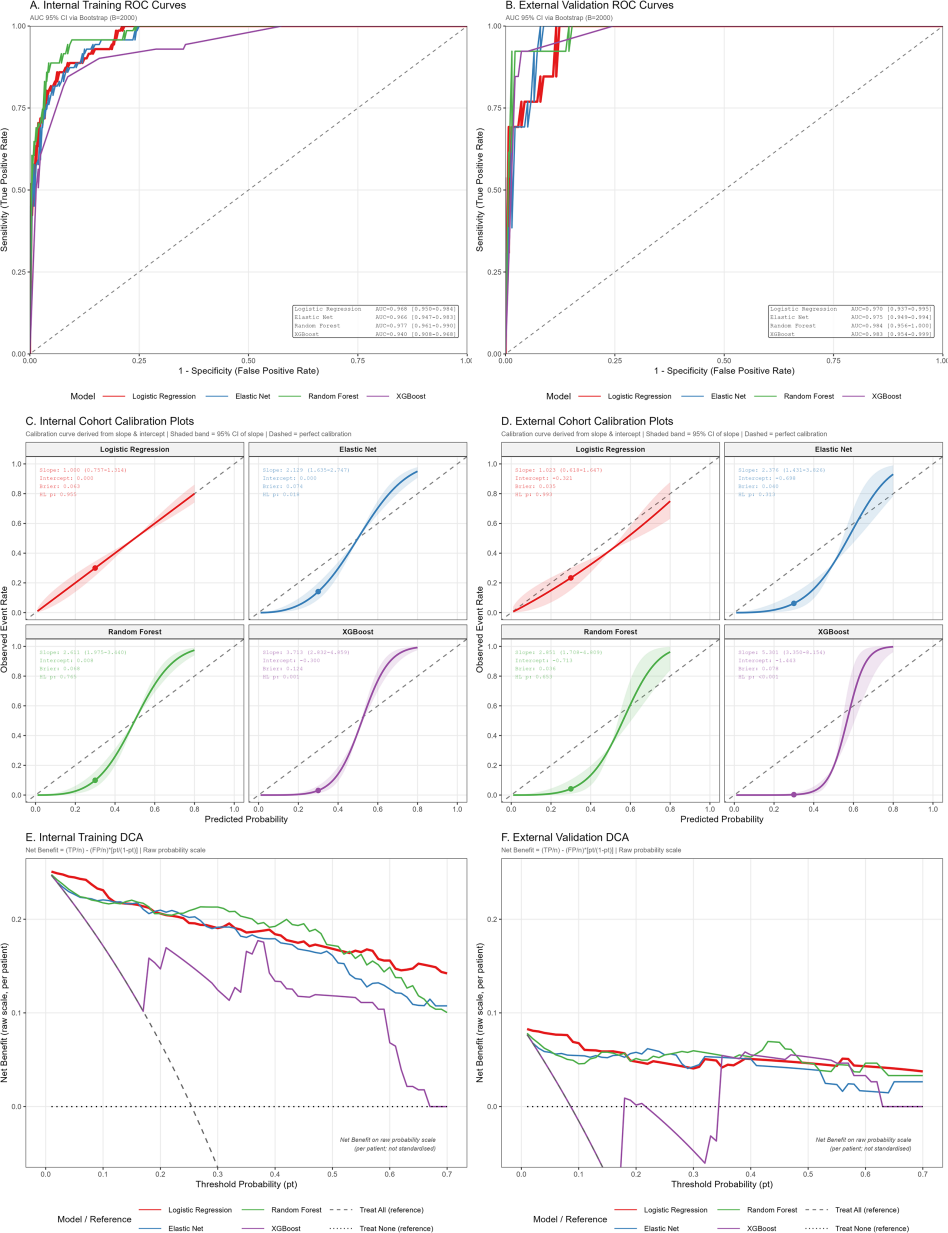
Evaluation of predictive performance for the four machine learning models. Six-panel figure showing **(A, B)** receiver operating characteristic curves, **(C, D)** calibration plots, and **(E, F)** decision curve analyses for logistic regression, elastic net, random forest, and XGBoost models in the derivation and temporal validation cohorts. AUC, area under the receiver operating characteristic curve; CI, confidence interval; DCA, decision curve analysis; ENet, elastic net; LR, logistic regression; RF, random forest; ROC, receiver operating characteristic; XGBoost, extreme gradient boosting.

All four models showed high discrimination in internal testing, with area under the ROC curve values ranging from 0.940 to 0.977. RF achieved the best overall internal performance (area under the ROC curve 0.977; 95% confidence interval 0.962–0.991), with accuracy 0.918, sensitivity 0.958, specificity 0.904, and F1-score 0.855 (**Figure 3**). LR and ENet also performed strongly (area under the ROC curve 0.968 and 0.966, respectively), whereas XGBoost had slightly lower discrimination (area under the ROC curve 0.940).

Model hyperparameters were tuned using grid search with cross-validation (GridSearchCV) in scikit-learn, and the optimized hyperparameters are summarized in **Table 2**. Each final model was then refitted using the tuned hyperparameters and the eight selected predictors.

**Table 2:** Performance comparison of the machine learning models in the internal validation cohort.

Model	AUC (95% CI)	Accuracy	Sensitivity	Specificity	PPV	NPV	F1
Internal validation cohort							
Logistic regression	0.968 (0.951–0.986)	0.907	0.887	0.913	0.778	0.960	0.829
Elastic net	0.966 (0.947–0.984)	0.889	0.930	0.875	0.717	0.973	0.810
Random forest	0.977 (0.962–0.991)	0.918	0.958	0.904	0.773	0.984	0.855
XGBoost	0.940 (0.910–0.970)	0.896	0.845	0.913	0.769	0.945	0.805

In the temporal external validation cohort, discrimination remained high. The area under the ROC curve (95% confidence interval) was 0.970 (0.942–0.999) for LR, 0.975 (0.952–0.998) for ENet, 0.984 (0.961–1.000) for RF, and 0.983 (0.959–1.000) for XGBoost (**Figure 3**). Additional performance metrics are provided in **Table 3**, and decision-curve analysis is shown in **Figure 3**.

**Table 3:** Performance comparison of the machine learning models in the external validation cohort.

Model	AUC	95% CI	Accuracy	Sensitivity	Specificity	PPV	NPV	F1
External validation cohort								
Logistic regression	0.970	0.942–0.999	0.894	1.000	0.884	0.448	1.000	0.619
Elastic net	0.975	0.952–0.998	0.927	1.000	0.920	0.542	1.000	0.703
Random forest	0.984	0.961–1.000	0.980	0.923	0.986	0.857	0.993	0.889
XGBoost	0.983	0.959–1.000	0.960	0.923	0.964	0.706	0.993	0.800

Across cohorts, RF showed the most consistent advantage, with the highest area under the ROC curve and a favorable F1-score. RF also maintained high recall, reducing the likelihood of missing patients who ultimately underwent major amputation—an important consideration for inpatient escalation of care. Therefore, subsequent explainability analyses focused on the RF model.

### 3.4 Calibration and decision-curve analysis

In the derivation cohort, predicted risks from all four models (LR, ENet, RF, and XGBoost) were generally concordant with observed event rates, with calibration curves close to the ideal (36)-degree line. Quantitative calibration metrics are summarized in **Table 4**. LR and ENet tracked the ideal line slightly better across most of the risk range (e.g., LR calibration slope 1.000), whereas RF and XGBoost were somewhat conservative at higher predicted risks but did not show clear systematic miscalibration, as supported by acceptable goodness-of-fit Hosmer-Lemeshow p = 0.765 for the RF model The 95% confidence intervals (CIs) for the AUCs were calculated using the bootstrap method with 2,000 resamples with replacement to provide robust interval estimates.

**Table 4:** Calibration metrics of the machine learning models in the internal validation cohort and temporal external validation cohort.

Cohort	Model	Brier score	Calibration intercept	Calibration slope (95% CI)	Hosmer-lemeshow test (p-value)
Internal Validation	Logistic Regression	0.063	0.000	1.000 (0.757 - 1.314)	0.955
	Elastic Net	0.074	0.000	2.129 (1.635 - 2.747)	0.018
	Random Forest	0.068	0.008	2.611 (1.975 - 3.440)	0.765
	XGBoost	0.124	-0.300	3.713 (2.832 - 4.859)	0.001
External Validation	Logistic Regression	0.035	-0.321	1.023 (0.618 - 1.647)	0.993
	Elastic Net	0.040	-0.698	2.376 (1.431 - 3.826)	0.313
	Random Forest	0.036	-0.713	2.851 (1.708 - 4.809)	0.653
	XGBoost	0.078	-1.443	5.301 (3.350 - 8.154)	<0.001

CI, confidence interval. Ideal calibration is indicated by a calibration intercept of 0 and a calibration slope of 1. Lower Brier scores indicate better overall probabilistic accuracy.

In the temporal validation cohort, calibration curves were more variable and confidence intervals widened at the highest risk quantiles; however, overall agreement between predicted and observed risk remained acceptable, with the primary RF model maintaining a highly competitive Brier score of 0.036 (**Table 4**).

### 3.5 Explainability results

**Figure 4:**
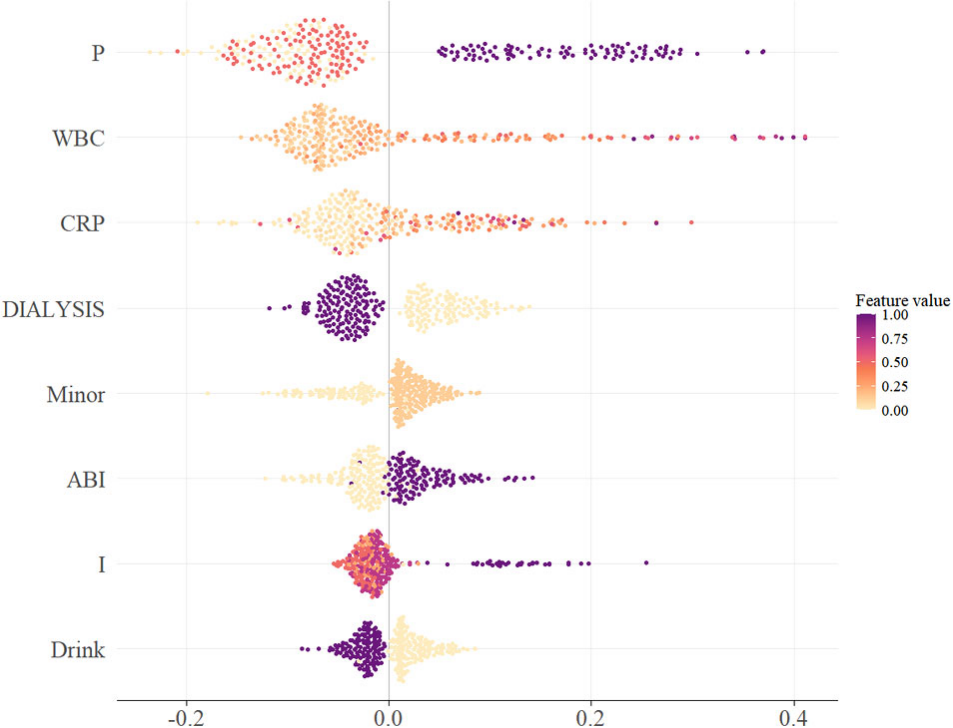
SHAP summary plot of key predictors in the random forest model. SHAP summary plot ranking the selected predictors in the random forest model, with each point representing one patient and colored according to feature value, showing the direction and magnitude of each predictor’s contribution to in-hospital major amputation risk.ABI, ankle-brachial index; CRP, C-reactive protein; Minor, prior minor amputation history; P, perfusion grade; RF, random forest; SHAP, Shapley additive explanation; WBC, white blood cell count.

We performed explainability analyses for the selected RF model. Global feature importance identified PEDIS perfusion grade P as the most influential predictor, followed by white blood cell count, C-reactive protein, maintenance dialysis, prior minor amputation, ankle–brachial index, PEDIS infection grade I, and alcohol use (**Figure 4**). The SHAP summary plot indicated that worse perfusion, higher inflammatory markers, abnormal ankle–brachial index, dialysis, and more severe infection increased predicted major amputation risk, whereas better perfusion and lower inflammatory activity decreased risk.

**Figure 5:**
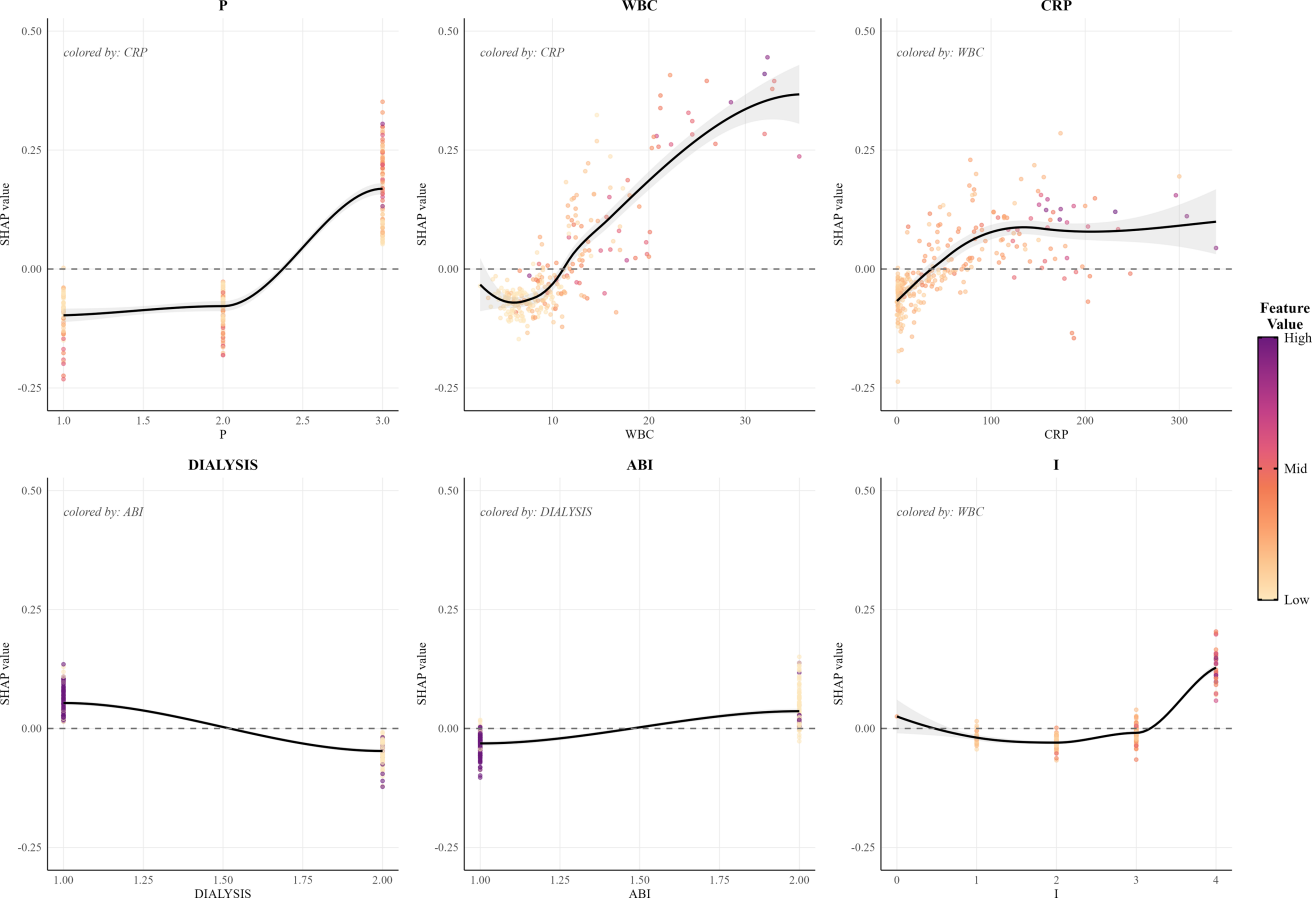
SHAP dependence plots and feature interactions of key predictors in the random forest model. Multi-panel SHAP dependence plots illustrating nonlinear relationships and feature interactions for key predictors, including perfusion grade, white blood cell count, C-reactive protein, dialysis status, ankle-brachial index, and infection grade, in the random forest model. ABI, ankle-brachial index; CRP, C-reactive protein; Dialysis, dialysis status; I, infection grade; RF, random forest; SHAP, Shapley additive explanation; WBC, white blood cell count.

SHAP dependence plots suggested nonlinear effects and potential thresholds for key laboratory predictors. For example, C-reactive protein above approximately 40–60 mg/L and white blood cell count above roughly 10×109/L were associated with progressively larger positive SHAP values, consistent with a steep rise in predicted risk beyond these ranges. Severe perfusion impairment P3 combined with abnormal ankle–brachial index values clustered at high SHAP values, suggesting additive effects of ischemia and arterial disease. Dialysis status showed predominantly positive SHAP values, indicating higher baseline risk among patients receiving maintenance dialysis. SHAP dependence plots for representative variables illustrated nonlinear feature effects and interactions how perfusion, inflammatory markers, dialysis, and other predictors jointly determined individualized risk estimates (**Figure 5**).

## 4 Discussion

### 4.1 Overall model performance and clinical utility

In this single-center retrospective cohort of hospitalized patients with diabetic foot ulcers (DFU), we developed several machine learning (ML) models to predict in-hospital major lower-extremity amputation using routinely available admission data, and selected a random forest (RF) model as the primary model based on its overall performance and explainability. All four algorithms (RF, XGBoost, LR and ENet) achieved high discrimination in the derivation cohort, with AUC values exceeding 0.94, and the RF model showed the most favorable balance between AUC, accuracy, sensitivity and F1 score. In the temporal external validation cohort, discrimination declined only modestly and calibration remained acceptable, indicating that the model retained reasonable performance when applied to patients admitted in a different time period. Decision-curve analysis further demonstrated that across a broad range of clinically relevant threshold probabilities, RF provided greater net benefit than either treating all or treating no patients, supporting its potential clinical utility as an early risk-stratification tool.

These findings are broadly in line with previous ML-based studies on DFU-related amputation, which have reported AUCs in the range of 0.80–0.90 for predicting major amputation or composite adverse outcomes.we applied oversampling techniques to correct for the imbalance between amputation and non-amputation cases; however, this was done on a cohort of consecutively hospitalized DFU patients from a tertiary referral center, covering a broad spectrum from moderate to severe disease. The relatively high proportion of severe ischemia and amputation events provided a more informative baseline dataset for model training, and oversampling was combined with rigorous feature selection and cross-validation to mitigate overfitting. In addition, temporal external validation was used to test model performance in an independent time-based cohort, thereby reducing the likelihood of overly optimistic estimates. Taken together with previous literature, our results further support the notion that ML models built on routinely collected clinical and laboratory variables can provide robust prognostic information for major amputation in DFU, provided that class imbalance is handled carefully and validation is appropriately performed.

### 4.2 Machine-learning algorithms and explainability

A methodological strength of this study is the parallel comparison of multiple ML algorithms under the same feature set following LASSO-based feature selection. Although differences in AUC between RF, XGBoost and penalised logistic regression were modest, RF consistently offered the best trade-off between discrimination and overall classification performance. This is consistent with the known strengths of tree-based ensemble approaches in capturing complex, non-linear associations and higher-order interactions—features that are highly relevant in DFU, where limb ischemia, infection, renal dysfunction and systemic inflammation interact in a multifactorial manner. At the same time, the performance gap between RF and simpler models remained relatively small, suggesting that, when predictors are carefully selected and clinically meaningful, traditional regression models remain viable alternatives in resource-limited settings or where technical support for advanced ML is not readily available.

Unlike many earlier “black-box” ML models, our work placed particular emphasis on explainability. Using SHapley Additive exPlanations (SHAP), we were able to decompose the RF model’s predictions into patient-level contributions from each feature, thereby aligning probabilistic outputs with clinical reasoning. This is particularly important for building trust among clinicians and for integrating ML tools into multidisciplinary decision-making pathways. Rather than merely stating that a patient is “high risk”, the model can show that the elevated risk is mainly driven, for example, by P3 perfusion, very high CRP and dialysis, whereas another high-risk patient’s prediction may be dominated by extensive infection and moderate ischemia but preserved renal function.

### 4.3 Key predictors and pathophysiological interpretation

Interpretability analyses consistently indicated that PEDIS perfusion grade P was the dominant driver of in-hospital major amputation risk, followed by WBC, CRP, dialysis status, ABI, PEDIS infection grade I, and history of minor amputation. Albumin and selected coagulation-related indices (e.g., fibrinogen and D-dimer) also contributed. Collectively, these predictors map onto four pathophysiological domains: (1) limb ischemia, (2) local/systemic inflammation, (3) renal dysfunction and an adverse metabolic milieu, and (4) chronic tissue injury and nutritional status—highlighting that limb outcomes in DFU are shaped by both local wound severity and systemic vulnerability. In interpreting these predictors, potential confounding and collinearity should also be considered. Clinically related variables such as dialysis status, inflammatory markers, nutritional indices, and measures of limb severity may cluster within the same high-risk patients and therefore may not represent fully independent pathophysiologic pathways. For example, dialysis is often associated with greater systemic illness, immune dysfunction, vascular compromise, and chronic inflammatory burden, while markers such as white blood cell count or C-reactive protein may capture both local infection severity and broader systemic deterioration. Accordingly, the contribution of these variables in our model should be interpreted primarily in predictive rather than causal terms. Their importance reflects the information they provide within the multivariable prediction framework, not necessarily a stand-alone etiologic effect.

First, the central role of perfusion impairment aligns closely with extensive evidence that critical limb ischemia is one of the most decisive determinants of limb loss in DFU (37–39). In the SHAP interpretability analysis, cases with PEDIS P3 severe ischemia together with an abnormal ABI (particularly markedly reduced ABI values) clustered within the highest-risk region of the SHAP space, indicating that profound lower-limb hypoperfusion is a key state driving in-hospital major amputation risk. This observation is well supported by external evidence: the IWGDF guideline on PAD in people with DFU highlights very low hemodynamic indices such as ABI <0.5 or ankle pressure <50 mmHg as markers of poor healing potential and increased amputation risk, and recommends prompt vascular assessment with consideration of revascularisation (30, 37). IWGDF Guidelines Moreover, Brownrigg et al.’s systematic review of prognostic PAD markers in active DFU reported that markedly reduced ABI is associated with higher major amputation risk, further corroborating the dominant role of ischemia-related signals in our model.

Second, inflammatory biomarkers (WBC and CRP) emerged as strong predictive signals. SHAP dependence patterns suggested that when CRP rose above ~40–60 mg/L and WBC exceeded ~10×10^9^/L, SHAP values increased steeply, implying a disproportionate rise in major amputation risk beyond these ranges. This finding is in line with evidence from the existing literature, as meta-analyses have shown that elevated white blood cell (WBC) counts are not uncommonly associated with an increased risk of amputation among patients with diabetic foot ulcers, supporting inflammatory burden as a key driver of adverse limb outcomes (40). In addition, prior studies on major LEA in DFU repeatedly report that elevated CRP is associated with increased amputation risk, corroborating the biological plausibility of the “systemic inflammation → limb loss” pathway highlighted by our model (41, 42).

Third, dialysis status—used as a proxy for end-stage kidney disease—showed an overall positive SHAP distribution, indicating a higher baseline risk of major amputation. This aligns with large-scale evidence: nationwide cohort data in ESKD populations demonstrate that dialysis-related factors are closely associated with increased LEA risk, consistent with impaired wound healing capacity and accelerated vasculopathy in renal failure (43). In parallel, nutritional status also mattered. In our cohort, major amputation cases more frequently exhibited hypoalbuminemia; prior studies similarly report lower albumin as a risk factor for DFU-related amputation, plausibly reflecting reduced reparative reserve and an intertwined malnutrition–inflammation state that compromises tissue healing (3). Together, these findings underscore that beyond local wound phenotype, systemic vulnerability captured by renal dysfunction and the nutrition–inflammation axis can be decisive for limb-salvage outcomes (44).

Fourth, the effect of prior minor amputation was clearly context dependent. Although univariable comparisons suggested a higher prevalence of prior minor amputation in the non–major amputation group, SHAP values were heterogeneous, indicating that this variable should not be interpreted as uniformly “protective” or “harmful.”A history of minor amputation should not be viewed merely as a treatment label; rather, it represents a prior tissue-loss event and cumulative disease burden, often implying longstanding neuropathy, PAD/ischemia and/or infection, together with biomechanical changes that predispose to re-ulceration (36). The IWGDF risk stratification system similarly classifies prior lower-extremity amputation (including minor amputation) as a high-risk state requiring intensified surveillance. Importantly, minor amputation does not eliminate future risk: contemporary Medicare claims data indicate that roughly one-quarter of patients with concomitant DM/PAD and prior minor amputation progress to major amputation within 5 years, supporting a persistently high-risk trajectory (45). In exploratory stratified and interaction analyses, the association between prior minor amputation and in-hospital major amputation appeared to be modified mainly by dialysis status, ABI abnormality, inflammatory burden, and infection severity. Accordingly, in lower-risk physiological settings, prior minor amputation may reflect successful previous local control and an established care pathway; in contrast, in the presence of severe ischemia, dialysis dependence, marked inflammation, or severe infection, it may instead indicate advanced disease burden and limited limb-salvage reserve (46). Prior DFU studies likewise identify previous amputation history—alongside CRP, albumin and PAOD—as important predictors of major LEA, supporting this interpretation (47). These findings warrant formal evaluation of such interactions in larger multicenter cohorts.

These SHAP-derived patterns should be interpreted cautiously. The apparent increases in predicted risk observed around CRP levels of approximately 40–60 mg/L and WBC values above approximately 10 × 10^9/L were identified visually from dependence plots and should not be interpreted as formally estimated clinical thresholds. Rather, they represent exploratory, model-based indications of possible nonlinear risk acceleration. More rigorous quantitative approaches, such as generalized additive models or spline-based analyses, would be needed to confirm whether true inflection points exist and to better characterize the functional form of these relationships (48, 49). Likewise, the relationship between perfusion grade and ABI warrants more systematic evaluation (50–52). Although ABI is widely used to identify peripheral arterial disease, it may be falsely elevated in patients with diabetes because of medial arterial calcification and non-compressible vessels. This limitation may help explain discordance between ABI findings and clinically assessed perfusion severity in some patients (52–54). Accordingly, the observed interplay between ABI and perfusion grade in the SHAP analyses should be viewed as clinically suggestive rather than conclusive, and future studies should examine this interaction using formal interaction terms and more flexible nonlinear modeling strategies.

### 4.4 Clinical implications

This study has several important clinical implications. First, the models were built exclusively on information obtainable within 24 hours of admission—demographics, comorbidities, PEDIS components, ABI and routine laboratory parameters—without requiring advanced imaging or specialized biomarkers. This makes them particularly suitable for early risk stratification in routine inpatient settings, including hospitals with limited resources. Second, the RF model, supported by SHAP-based explanations, can provide both numerical risk estimates and transparent explanations of the main risk drivers for each patient, which may facilitate multidisciplinary decision-making involving endocrinologists, vascular surgeons, foot and ankle surgeons, nephrologists and rehabilitation teams. High-risk patients could be prioritized for urgent vascular imaging, timely consideration of revascularization, aggressive infection control, optimization of nutritional and renal status, and closer monitoring. Third, decision-curve analysis indicated that model-guided strategies offer higher net benefit than “treat-all” or “treat-none” approaches across clinically relevant thresholds, suggesting that integrating this model into electronic health records or bedside decision-support systems could improve the allocation of limited surgical and vascular resources. Finally, in a practical implementation setting, the model could be triggered shortly after admission using routinely available clinical and laboratory data extracted from the electronic health record, supplemented by structured bedside inputs where necessary. Risk estimates would ideally be accompanied by concise SHAP-based explanations showing the main factors driving risk for an individual patient, thereby supporting multidisciplinary decision-making rather than replacing clinician judgment. Ethical considerations should also be kept in mind when applying amputation-risk prediction models. In particular, high predicted risk should not lead to therapeutic nihilism, premature acceptance of amputation, or reduced effort toward limb salvage. Instead, model outputs should be used to support earlier multidisciplinary evaluation, more equitable allocation of specialist resources, and more transparent shared decision-making, while preserving individualized clinical judgment and attention to patient preferences.

### 4.5 Strengths, limitations and future directions

This study has several strengths. It focuses on an important yet challenging outcome—major amputation in hospitalized DFU patients—and uses routinely collected admission data to develop an explainable ML model that was temporally validated. It systematically compares multiple algorithms after structured feature selection and incorporates SHAP to bridge algorithmic predictions with clinical reasoning.

However, several limitations should be acknowledged. First, this investigation was conducted at a single center with a retrospective design and a relatively modest sample size, particularly in the temporal validation cohort, which may limit the precision of performance estimates and may constrain the extent to which the results can be generalized. Second, as a tertiary referral center, our hospital receives many severe DFU cases; therefore, the model may perform differently in populations with milder disease. In addition, patients with active malignancy were excluded, which improved cohort clinical consistency but may further limit generalizability to medically more complex DFU populations encountered in some institutions. The prevalence of this exclusion has now been reported in the cohort screening process. Third, although temporal validation was performed using an independent cohort from 2024, this cohort was drawn from the same institution and was separated from the derivation cohort by a four-year interval. During this period, changes in clinical practice—such as revascularization strategies, antibiotic stewardship, wound-care pathways, referral thresholds, or perioperative decision-making—may have occurred and could have influenced major amputation risk independently of baseline patient characteristics. Because we did not formally collect institution-level process measures to quantify these changes, their potential impact on model performance cannot be excluded. Therefore, the temporal validation results should be interpreted as reflecting both patient-level generalization across time and the possibility of practice-pattern drift across calendar periods. While the model retained predictive value in a later non-overlapping cohort, this finding should be interpreted as preliminary evidence of temporal robustness within the same clinical environment rather than definitive proof of stability across evolving practice settings. In addition, the relatively small size of the temporal validation cohort may limit the precision of performance estimates, particularly for calibration, and may not fully represent the full spectrum of hospitalized DFU presentations encountered in routine clinical practice. Fourth, we focused exclusively on in-hospital major amputation and did not capture long-term outcomes such as post-discharge amputations, mortality, or quality of life; therefore, the model should be viewed primarily as an early in-hospital decision-support tool rather than a long-term prognostic instrument. Moreover, because time to amputation was not modeled separately, the model’s practical predictive window should be interpreted with caution. Finally, in community hospitals or lower-acuity settings, the case mix may include fewer patients with advanced ischemia, severe infection, dialysis dependence, or extensive tissue loss, and clinical pathways for vascular intervention, infectious disease consultation, wound care, and amputation decision-making may also differ. Under such conditions, both baseline event rates and the relative contribution of individual predictors may shift, potentially affecting model discrimination and calibration. In addition, although ABI was dichotomized at <0.9 for pragmatic clinical interpretability and consistency with common vascular risk stratification, this approach may have reduced information on PAD severity, particularly at lower ABI values. For this reason, local recalibration or model updating may be necessary before adoption in institutions with substantially different patient populations or care pathways.

Future work should also explore the incorporation of dynamic clinical trajectories and richer imaging and microbiological data, and should apply spline-based or generalized additive modeling approaches to formally evaluate potential nonlinear biomarker-risk relationships and interaction effects, particularly for inflammatory markers and vascular assessment variables such as ABI and perfusion grade. Future studies may also compare the present framework with LASSO-based prognostic nomogram approaches that have been applied in other clinical settings (55). Integration into electronic health record systems and prospective implementation studies will be crucial steps toward translating this work into routine practice. Such studies should evaluate practical issues including alert thresholds, timing of model activation, structured variable capture, explanation display, clinician acceptance, and the impact of model-guided strategies on real-world clinical decision-making, resource allocation, and limb-salvage outcomes.

## 5 Conclusion

In this single-center retrospective cohort study, we developed and temporally validated an explainable admission-data model to predict in-hospital major amputation among hospitalized patients with diabetic foot ulcer. Across internal testing and temporal validation, the random forest model provided high discrimination and clinically useful net benefit. Explainability analyses indicated that worse limb perfusion (PEDIS perfusion grade and ankle–brachial index), higher inflammatory burden (white blood cell count and C-reactive protein), maintenance dialysis, more severe infection, and prior minor amputation were associated with higher predicted major amputation risk. This framework may support early inpatient risk stratification and prioritization of multidisciplinary limb-salvage care; future multicenter prospective validation and evaluation of clinical impact are warranted.

## Data Availability

The original contributions presented in the study are included in the article/**Supplementary Material**. Further inquiries can be directed to the corresponding author.

## Supplementary material

Supplementary Table 1 Final hyperparameter configurations for the evaluated machine learning models.
