# Supplementary material for: Interpretable machine learning for predicting major amputation risk in hospitalized diabetic foot ulcer patients: a single-center study with temporal external validation
Source: Front Endocrinol (Lausanne). 2026 May 22;17:1821550. doi: 10.3389/fendo.2026.1821550 (PMC13236543; doi:10.3389/fendo.2026.1821550)
Supplement: Supplementary Table 1 — Final hyperparameter configurations for the evaluated machine learning models. [file DataSheet1.docx]

Supplementary Table 1. Final hyperparameter configurations for the evaluated machine learning models.

| **Model** | **Algorithm/Engine** | **Hyperparameters and Final Values** | **Note on Configuration Strategy** |
| --- | --- | --- | --- |
| Logistic Regression | glm (stats) | Penalty = None | Standard maximum likelihood estimation; applied after LASSO feature selection. |
| Elastic Net | glmnet | Penalty (λ) = 0.05  Mixture (α) = 0.5 | Equal blend of Ridge (L2) and Lasso (L1) penalties to manage multicollinearity. |
| Random Forest | ranger | Trees = 500  Min Node Size (min_n) = 40 | High minimum node size explicitly selected via cross-validation to aggressively prevent overfitting in the modestly sized training set. |
| XGBoost | xgboost | Trees (nrounds) = 30  Max Depth = 1  Learning Rate (η) = 0.05 | Heavily regularized (shallow stumps and low learning rate) to ensure generalizability and avoid fitting noise. |
